# *E. coli* Endotoxin Modulates the Expression of Sirtuin Proteins in PBMC in Humans

**DOI:** 10.1155/2013/876943

**Published:** 2013-11-19

**Authors:** Angela Storka, Gerhard Führlinger, Martin Seper, Lisa Wang, Michael Jew, Asha Leisser, Michael Wolzt

**Affiliations:** ^1^Department of Clinical Pharmacology, Medical University of Vienna, Währinger Gürtel 18-20, 1090 Vienna, Austria; ^2^Department of Internal Medicine I, Institute of Cancer Research, Medical University of Vienna, Währinger Gürtel 18-20, 1090 Vienna, Austria

## Abstract

*Background*. Sirtuin (SIRT) proteins are class I histone deacetylases displaying gene regulatory functions in inflammatory, cancer, and metabolic diseases. These SIRT actions involve the nuclear factor **κ**B and its inhibitor I**κ**B pathway. However, the regulation of SIRT *in vivo* is still unclear. *Material and Methods*. In a human endotoxemia model, 20 healthy male subjects received an intravenous bolus of 2 ng/kg body weight *Escherichia coli* endotoxin (LPS). SIRT expression was investigated in peripheral blood mononuclear cells (PBMC) with qPCR and Western blot before and 3 hours, 6 hours, and 24 hours after LPS challenge. Additionally, SIRT regulation was studied *in vitro* in cultivated PBMC after incubation with 20 ng/mL LPS. *Results*. A downregulation by >40% of SIRT1 mRNA was detectable 3 hours after LPS and of SIRT3 mRNA 6 hours after LPS. SIRT3, I**κ**B**α**, and I**κ**B-**β** protein expressions were decreased 3 and 6 hours after LPS. SIRT2 mRNA or protein expression did not change following LPS. These findings were consistent *in vitro* and associated with augmented phosphorylation of I**κ**B-**β**. *Discussion*. In this *E. coli* endotoxemia model, SIRT1 and SIRT3 mRNA expressions in PBMC in humans were reduced after LPS challenge. This suggests that SIRT may represent an inflammatory target protein *in vivo*.

## 1. Background

Nicotinamide phosphoribosyltransferase (Nampt), also known as pre-B-cell colony-enhancing factor 1 (PBEF1) or visfatin, is an enzyme in humans that catalyzes the condensation of nicotinamide with 5-phosphoribosyl-1-pyrophosphate to yield nicotinamide mononucleotide, which is required in the biosynthesis of nicotinamide adenine dinucleotide (NAD^+^). As a pivotal component of an NAD^+^ salvage pathway, Nampt is also a regulator of the activity of NAD^+^-dependent enzymes, such as class III histone/protein deacetylases (HDAC). Within this group are Sirtuin proteins, of which seven isoforms (SIRT1–7) have so far been identified in humans. Sirtuins possess a wide range of regulatory functions and are implicated in inflammatory diseases, cancer, and aging [1]. The founding Sirtuin member, SIRT1, is associated with stress resistance, metabolism, apoptosis, senescence, differentiation, and aging [1] by directly regulating gene expression through its epigenetic activity on histones and gene silencing [2]. SIRT2, a tubulin deacetylase, functions as a mitotic checkpoint protein [3] and mediates mitotic cell death in response to DNA damage [4].

Recently, it has also been shown that SIRT1 and SIRT2 exert anti-inflammatory effects by deacetylating the RelA/p65 subunit of nuclear factor *κ*B (NF-*κ*B) at lysine 310, which inhibits NF-*κ*B DNA transcription activity involved in regulating proinflammatory cytokines [5–8]. In another study, Gao and Ye showed that c-Jun transcriptional activity can be downregulated by SIRT1 *in vitro*. In combination with c-Fos, c-Jun makes up the dimeric transcription factor AP-1, a major regulator of immune cell function [9]. In an animal study, SIRT1^−/−^ mice displayed increased T-cell responsiveness and were predisposed to develop autoimmune disorders [10]. Finally, Yang et al. demonstrated that SIRT1 activators suppressed the inflammatory response in an LPS-induced inflammation mouse model by deacetylation of p65 and inhibition of NF-*κ*B activity [11].

SIRT3 is localized in the mitochondria and plays a role in mitochondrial metabolism, reactive oxygen species (ROS) production, and genome stability. Koyama et al. recently showed that SIRT3 modulates activation of the mitogen-activated protein kinase- (MAPK-) NF-*κ*B pathway. The MAPK-NF-*κ*B pathway regulates cell proliferation, differentiation, motility, and survival via various extracellular stimuli. In SIRT3 overexpressing cells, phosphorylation of ERK1/2 and p38 and degradation of I*κ*B-*α* are enhanced, and ROS levels are decreased [12].

Small doses of intravenously administered lipopolysaccharide (LPS) lead to acute inflammatory responses that are qualitatively similar to those in the early stages of sepsis. Changes in systemic hemodynamics, endothelial function, permeability, pulmonary gas exchange, and ventricular function occur within 3 hours of intravenous administration of LPS endotoxin to healthy subjects [13–16]. A diversity of inflammatory mediators is released after LPS challenge, including proinflammatory cytokines (e.g., IL-8, tumor necrosis factor-alpha, IL-1 beta, and IL-6) [17–19].

Shen et al. demonstrated that LPS stimulation of murine macrophages (RAW 264.7) downregulates SIRT1 accompanied by increased acetylation of RelA/p65 and enhanced NF-*κ*B transcription activity *in vitro* [20]. However, *in vivo* studies characterizing the expression of SIRT in immune-competent cells and their regulation following immunological stimulation are not available. Accordingly, the present study was aimed to examine Sirtuin expression profiles in peripheral blood mononuclear cells (PBMC) following systemic LPS administration in healthy humans to clarify the involvement and potential roles of SIRT in the systemic inflammatory response of humans.

## 2. Material and Methods

The study was approved by the Ethics Committee of the Medical University Vienna (EK725/2007) and conforms to the principles outlined in the Declaration of Helsinki, including current revisions and the Good Clinical Practice guidelines. According to the study protocol, 20 healthy male subjects aged from 19 to 40 years (mean age 29 ± 6) were included. Following a complete health examination, which included physical examination, electrocardiogram, and laboratory screening, the subjects received intravenously 2 ng/kg body weight of *E. coli* LPS endotoxin (US Standard Reference Endotoxin; NIH-CC, Bethesda, MD, USA) for over 3 minutes to induce a systemic inflammatory response. Venous blood samples were collected at baseline and 3, 6, and 24 hours after LPS administration for PBMC isolation.

### 2.1. PBMC Isolation from Whole Blood

PBMC were harvested using Ficoll density gradient centrifugation. Ficoll-Paque PLUS (GE Healthcare Life Sciences, Chalfont St Giles, UK) was overlaid with 5 mL EDTA blood and centrifuged at room temperature for 20 minutes at 500 g. Cells were then collected, washed twice with phosphate buffered saline (PBS), and harvested for Western blot or PCR analysis.

### 2.2. PBMC Cultivation

For *in vitro* experiments PBMC were isolated from healthy donors (*n* = 3) as mentioned above from 56 mL EDTA blood. PBMC were resuspended in RPMI-1640 (Gibco, Grand Island, NY, USA) containing 1% L-glutamine, 100 *μ*g/mL penicillin G, 100 *μ*g/mL streptomycin, and 5 *μ*g/mL Fungizone (Gibco) supplemented with 10% heat inactivated fetal calf serum (Sigma) and seeded in 16 mm culture wells. PBMC were incubated for further Western blot analysis the following day with 20 ng/mL *E. coli* LPS 0111:B4 (Sigma, St. Louis, MI, USA) for 3 h, 6 h, or 24 h and compared to unstimulated cells.

### 2.3. Western Blot Analysis

To assess the protein expression of SIRT, time-course Western blot analysis of the proteins SIRT1, SIRT2, SIRT3, PBEF, I*κ*B-*β*, I*κ*B*α*, and phospho-I*κ*B-*α* was performed. PBMC proteins were extracted in 60 *μ*L phosphorolysis buffer containing 100 mM NaCl, 0.1% SDS, 1% Nonidet P40, 50 mM Tris, pH 7.4, 10 mM EDTA, 10 mM p-nitrophenolphosphate, 40 mM b-glycerophosphate, and Complete Protease Inhibitor Cocktail Tablets (Roche, Mannheim, Germany). The amount of soluble proteins was quantified by modified Bradford assay (Bio-Rad, Richmond, CA, USA). Total cell lysates (15 *μ*g/lane) were separated by 12% SDS polyacrylamide gel electrophoresis and blotted onto PVDF membranes (GE Healthcare, UK). The membranes were blocked with 0.2% I-Block (Tropix, Bedford, MA, USA) in 1 M Tris-buffered saline containing 0.1% Tween-20 (Sigma, St. Louis, MI, USA) followed by overnight incubation at 4°C with the following primary antibodies: SIRT1, SIRT2, and SIRT3 (1 : 1000, Cell Signalling Technology, Beverly, MA, USA); I*κ*B-*β* (dilution 1 : 500, Delta Biolabs Muraoka Drive Gilroy, CA, USA); I*κ*B-*α* and phospho- I*κ*B-*α* (both 1 : 1000, Cell Signalling Technology, Beverly, MA, USA); PBEF (1 : 5000, Bethyl Laboratories Inc. Montgomery, TX, USA); and *β*-actin (1 : 5000, Sigma-Aldrich, St. Louis, MA, USA). The membranes were subsequently washed and incubated with HRP-conjugated goat anti-mouse or goat anti-rabbit immunoglobulins (1 : 5000, Santa Cruz Biotechnology Inc., Dallas, TX, USA). Relative band intensities were quantified using TotalLab Quant software (Nonlinear Dynamics, Newcastle upon Tyne, UK).

### 2.4. Real-Time qPCR

Total RNA was isolated from fresh whole blood samples using TRIzol reagent (Invitrogen, CA, USA) as per the manufacturer's instructions; the integrity of the RNA isolates was verified by gel electrophoresis. Aliquots of 2 *μ*g RNA samples were reverse transcribed into first strand cDNA with the RevertAid First Strand cDNA Synthesis Kit (Fermentas, MA, USA) using random hexamer primer in compliance with the standard protocol for PCR amplification. Real-Time qPCR was carried out using TaqMan Gene Expression Master Mix (Applied Biosystems, CA, USA), TaqMan Gene Expression Assays (Applied Biosystems, CA, USA), and 1 ng cDNA in a 20 *μ*L reaction mixture. PCR reactions were performed in a 7500 Fast Real-Time PCR System (Applied Biosystems, CA, USA) under the following conditions: an initial incubation at 50°C for 20s and then 95°C for 10 min, followed by 40 cycles of 15 s at 95°C and 1 min at 54°C.

Threshold cycle ΔΔ-Ct values for the SIRT1, SIRT2, and SIRT3 genes and the B2 M housekeeping gene were determined in triplicate. Relative quantification of RNA was calculated by the ΔΔ-Ct method. Samples with cDNA omitted were used as negative controls.

### 2.5. Statistical Analysis

Datasets were expressed as mean ± SD and presented with descriptive statistics. Since the data distribution was skewed, nonparametric tests were performed using SPSS software (IBM, NY, USA). After assessing Friedman-ANOVA analyses, the nonparametric Wilcoxon test was used to compare within groups. Statistical significance was defined using a *P* value <0.05.

## 3. Results

Following LPS challenge, transient flu-like symptoms were observed with an increase in body temperature from 36.1 ± 0.4°C to 37.1 ± 0.5°C (*P* < 0.01) after 3 hours and to 36.9 ± 0.4°C after 6 hours (*P* < 0.01). Leukocyte cell count significantly increased from 7.3 ± 1.4 G/L at baseline to 9.8 ± 2.7 G/L (*P* < 0.01) 3 hours after LPS and to 11.2 ± 1.4 G/L (*P* < 0.01) 6 hours after LPS, respectively.

### 3.1. Sirtuin Expression

**Figure 1:**
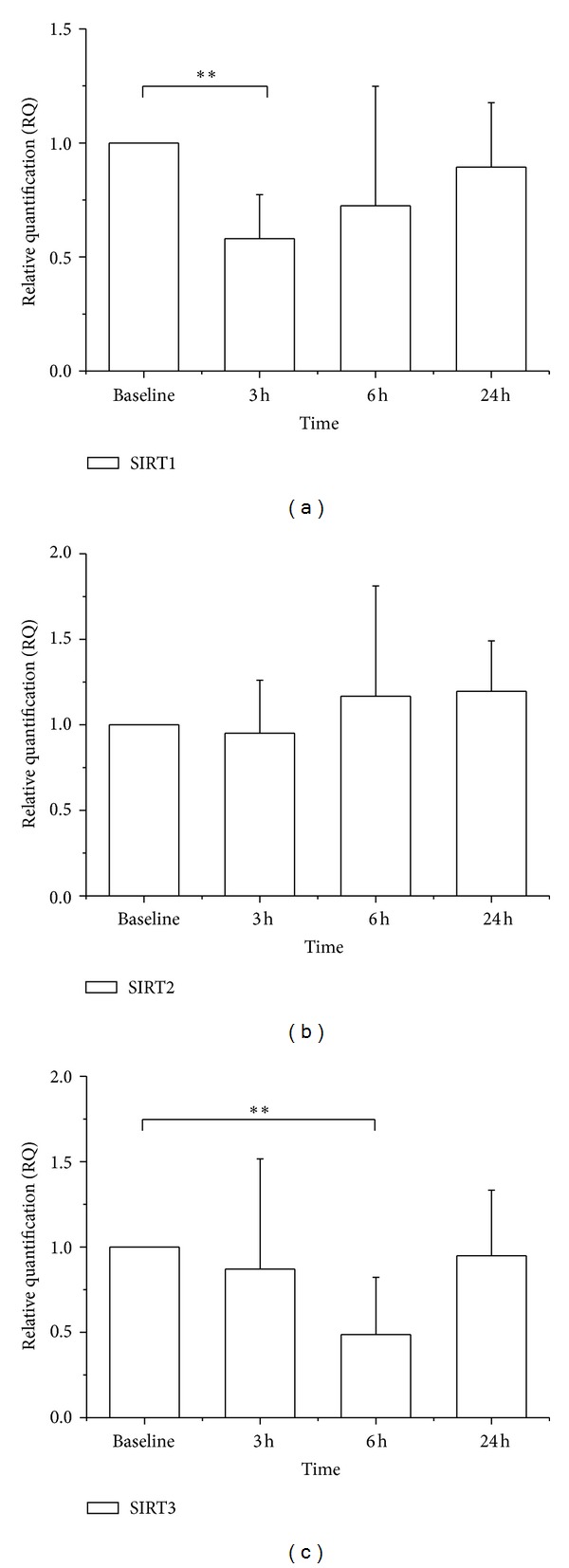
SIRT1, SIRT2, and SIRT mRNA expressions in PBMC following LPS administration. (a) SIRT1 mRNA was decreased 3 hours after LPS challenge (RQ 0.58; *P* < 0.01). (b) SIRT2 mRNA was not altered within 24 hours after LPS. (c) SIRT3 mRNA was decreased 6 hours after LPS challenge (RQ 0.49; *P* < 0.01). Results are mean ± SD (*n* = 7).

**Figure 2:**
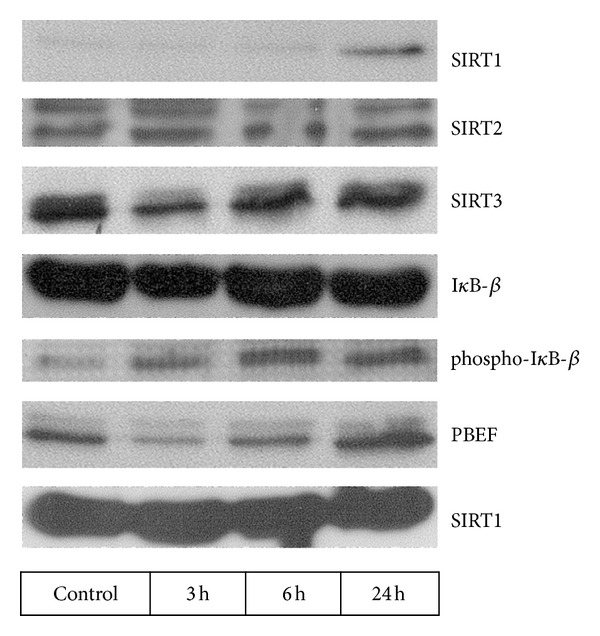
Protein expression of SIRT1, SIRT2, SIRT3, I*κ*B-*β*, phospho-I*κ*B-*β*, PBEF, and *β*-actin in isolated PBMC incubated with LPS. An upregulation of SIRT1 protein expression was detected 24 hours following LPS. SIRT2 protein expression was unchanged. SIRT3 decreased 3 hours after LPS. I*κ*B-*β* expression was decreased 3 hours after LPS, and phosphorylated I*κ*B-*β* was upregulated following LPS administration. PBEF showed first a decrease after 3 and 6 hours and an increase after 24 hours following LPS.

LPS administration significantly decreased SIRT1 mRNA expression to 58  ±  19% of baseline after 3 hours (Figure 1(a)). SIRT1 mRNA expression returned to baseline levels after 24 hours. SIRT1 protein expression was not detectable with Western blot in PBMC. *In vitro* incubation of PBMC with LPS for 24 h resulted in enhanced SIRT1 protein expression (Figure 2).

SIRT2 mRNA and protein expressions did not alter significantly following LPS (Figure 1(b)). Likewise, *in vitro* incubation with LPS had no effect on SIRT2 protein expression (Figure 2).

**Figure 3:**
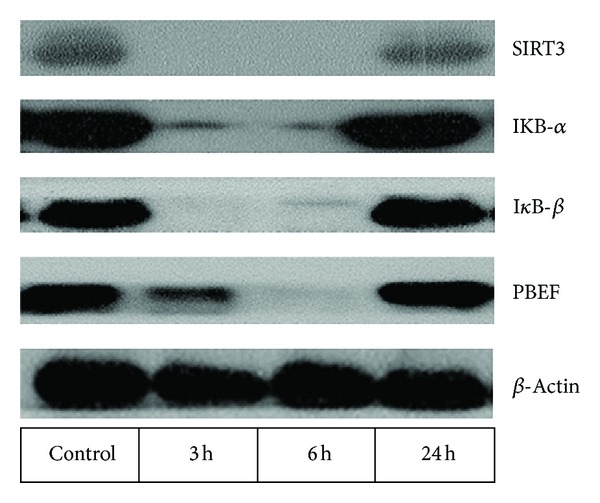
Protein expression of SIRT3, IKB-*α*, I*κ*B-*β*, PBEF, and *β*-actin in PBMC before and 3 h, 6 h, and 24 h after LPS administration in a healthy subject. A transient decrease in SIRT3, IKB-*α*, I*κ*B-*β*, and PBEF proteins was detectable.

SIRT3 mRNA expression decreased significantly 6 hours after LPS administration to 49 ± 33% of baseline and normalized after 24 hours *in vivo* (Figure 1(c)). SIRT3 protein in PBMC was also reduced 3 h and 6 h after LPS (Figure 3). Similarly, incubation of PBMC with LPS reduced SIRT3 protein expression after 3 and 6 hours *in vitro* (Figure 2).

### 3.2. PBEF Protein Expression

Systemic LPS administration decreased PBEF protein expression in PBMC after 3 and 6 hours, followed by a slight increase after 24 hours (Figure 3). This transient downregulation of PBEF protein expression was also noted when LPS was incubated with PBMC *in vitro* (Figure 2). 

### 3.3. I*κ*B-*β*, Phospho-I*κ*B*α*, and I*κ*B-*α* Protein Expressions

As expected, the NF-*κ*B pathway was activated by LPS. I*κ*B-*β* and I*κ*B-*α* proteins in PBMC of LPS-treated subjects were reduced after 3 h and 6 h. When incubated with LPS, the reduced expression of I*κ*B-*β* protein was paralleled by enhanced phospho-I*κ*B*α* protein (Figure 2). 

## 4. Discussion

An inflammatory response occurs after injurious stimuli, such as from bacterial infection, toxins, heat, or trauma, and is essential in the healing process. However, chronic or exaggerated inflammatory response can lead to tissue damage. Sirtuins have been documented to play pivotal roles in posttranslational modifications and regulation of the inflammatory response. We demonstrate here that SIRT protein expression in PBMC is altered in response to low dose systemic administration of LPS in healthy subjects.

*In vivo* SIRT1 mRNA expression was downregulated by approximately 40% of baseline levels in isolated PBMC within 3 hours of LPS challenge. Since expression of SIRT1 protein was trivial in PBMC in the subjects under study, a downregulation on protein level was not demonstrable. Nevertheless, a regulation of SIRT1 protein was evident when PBMC were incubated with endotoxin *in vitro*. In contrast to previous data, where a decrease in SIRT1 expression following LPS was reported [20], this was not seen in the present study. Again, this finding may be due to the fact that little if any SIRT1 protein is expressed in isolated PBMC under resting conditions. However, LPS caused a SIRT1 protein upregulation after 24 h incubation, which indicates a counterregulatory mechanism to the inflammatory stimulus. Further, our results for SIRT2 are at variance with data showing an inflammation-induced increase of SIRT2 *in vitro* and *in vivo* [8]. Of note, the cytokine TNF-*α* rather than endotoxin was used in these experiments, which could explain the discrepancy of findings.

In this human model of innate immune response, SIRT3 mRNA and protein expressions were substantially reduced 6 hours after LPS challenge, which was also confirmed in isolated PBMC. This novel evidence that SIRT3 expression is decreased by *E. coli* endotoxin *in vivo* again differs from other experimental settings, where increased SIRT3 protein was reported in mice microglia cells after 48 hours of incubation with *E. coli* LPS [21] or reduced SIRT3 expression in proximal tubular cells exposed to palmitate [12]. It is therefore unclear if our finding is specific for cell types, a time-related factor, or typical for the bacterial toxins used. It is presently unclear if these changes are adaptive as an acute response to *E. coli* LPS or may also be disease-causing due to dysregulation of SIRT expression in clinical conditions. Further studies are needed to investigate the role of SIRT in syndromes of severe systemic inflammation or similar simultaneous induction of pro- and anti-inflammatory processes.

PBEF functions as a nicotinamide phosphoribosyltransferase within the cell and catalyzes the rate-limiting step in the salvage pathway of NAD^+^ biosynthesis. NAD^+^ regulates cell energetic and NAD-dependent enzymes such as SIRT. Following systemic LPS challenge, PBEF protein expression initially decreased and was later upregulated relative to baseline. This is consistent with previous investigations that showed a peak expression of PBEF in isolated neutrophils and monocytes 10 hours after LPS stimulation [22].

Because class I histone deacetylases are postulated to be involved in the regulation of the NF-*κ*B pathway, we investigated I*κ*B expressions following LPS challenge. The diminished protein expression of I*κ*B-*α* and I*κ*B-*β* after LPS and increased expression of phospho-I*κ*B-*α* following incubation with LPS indicate an enhanced degradation of these proteins due to activation and nuclear translocation of NF-*κ*B. These results strengthen previous findings that class I histone deacetylases exert anti-inflammatory effects by deacetylating the RelA/p65 subunit of NF-*κ*B [5–8, 12, 20].

The present study suggests that SIRT1 and SIRT3 are regulated following an inflammatory stimulus with *E. coli* endotoxin *in vivo*. Our observations are in line with previous findings that SIRT proteins are relevant in modulating the NF-*κ*B pathway and suggest that SIRT activators could qualify as anti-inflammatory agents. Nevertheless, further studies are needed to show direct NF-*κ*B pathway modulating effects *in vivo*.
